# Molecular Imaging of Ulex Europaeus Agglutinin in Colorectal Cancer Using Confocal Laser Endomicroscopy (With Video)

**DOI:** 10.3389/fonc.2021.792420

**Published:** 2021-12-20

**Authors:** Weijun Wang, Shuxin Tian, Xin Jiang, Suya Pang, Huiying Shi, Mengke Fan, Zeyu Wang, Weiwei Jiang, Weiqian Hu, Xueyan Xiao, Rong Lin

**Affiliations:** ^1^ Department of Gastroenterology, Union Hospital, Tongji Medical College, Huazhong University of Science and Technology, Wuhan, China; ^2^ Department of Gastroenterology, National Health Commission (NHC) Key Laboratory of Prevention and Treatment of Central Asia High Incidence Diseases, the First Affiliated Hospital of Medical College, Shihezi University, Shihezi, China; ^3^ Department of Gastroenterology, the First Affiliated Hospital of Medical College, Shihezi University, Shihezi, China

**Keywords:** colorectal cancer, UEA-FITC, CLE, diagnostic efficacy, colorectal cancer imaging target

## Abstract

**Background and Study Aims:**

Previous studies have identified that colorectal cancer has different fucosylation levels compared to the normal colon. Ulex europaeus agglutinin-I (UEA-I), which specifically combines with α1-2 fucose glycan, is usually used to detect fucosylation levels. Therefore, we used confocal laser endomicroscopy (CLE) to investigate fluorescently labeled UEA-Fluorescein isothiocyanate (FITC) for detecting colonic cancer.

**Patients and Methods:**

We stained frozen mouse colon tissue sections of normal, adenoma, and adenocarcinoma species with UEA-FITC to detect fucosylation levels in different groups. White light endoscopy and endocytoscopy were first used to detect the lesions. The UEA-FITC was then stained in the mice and human colon tissues *in vitro*. The CLE was used to detect the UEA-FITC levels of the corresponding lesions, and videos were recorded for quantitation analysis. The diagnostic accuracy of UEA-FITC using CLE was evaluated in terms of sensitivity and specificity.

**Results:**

The UEA expression level in colorectal cancer was lower than that in normal intestinal epithelium. The fluorescence intensity ratio of UEA-FITC in colorectal cancer was significantly lower than that in normal tissue detected by CLE in both mice and humans. The combination of UEA-FITC and CLE presented a good diagnostic accuracy with a sensitivity of 95.6% and a specificity of 97.7% for detecting colorectal cancer. The positive and negative predictive values were 91.6% and 95.6%, respectively. Overall, 95.6% of the sites were correctly classified by CLE.

**Conclusions:**

We developed a new imaging strategy to improve the diagnostic efficacy of CLE by using UEA-FITC.

## Introduction

Combining cancer-specific molecular imaging technologies with optical imaging agents provides a novel technique for cancer detection. White light endoscopy (WLE) is the initial technique used to detect gastrointestinal tract tumors (1). Nevertheless, WLE has several disadvantages: it is insensitive to detecting multifocal and flat tumors, and is insufficient to judge tumor demarcation lines (2). These disadvantages may affect the detection and complete resection of tumors, which may influence the prognosis of cancer. Cancer-specific molecular imaging agents labeled by fluorescence may augment the distinction between tumors and adjacent normal or benign tissues. Consequently, development of molecular imaging agents that can provide enhanced tumor visualization is of great significance.

Colorectal cancer (CRC) is the third most commonly diagnosed malignancy and the fourth leading cause of cancer-related deaths worldwide (3). The global morbidity rate was high at 10.2%, and the mortality rate was high at 9.2% (4). The prognosis of colon cancer is closely related to its clinical stage. The 5-year survival rate for early colon cancer is 90%, and the 5-year survival rate for advanced colon cancer is 14% (5). Therefore, early diagnosis and treatment are essential to improve patient survival.

Colon tumors mostly develop from adenomatous polyps and look similar, but the treatment strategies are completely different (6, 7). Adenomatous polyps can be removed by cold snare polypectomy and endoscopic mucosal resection (EMR); colon tumors can be removed by endoscopic submucosal dissection (ESD), according to the invasion (6). In addition, when deep submucosal infiltration is suspected or confirmed, as the lymph node metastasis increases, the corresponding surgical operations must be performed, and adjuvant chemotherapy may be needed to eradicate the cancer (7). Clinically, appropriate treatment strategies should be selected according to the benign and malignant diseases of the colon.

As the main tool to detect, biopsy, and resect gastrointestinal tumors, WLE has several shortcomings, including difficulty in recognizing flat tumors and difficulty in estimating tumor boundaries (1). Over the past two decades, some novel endoscopic techniques have emerged as promising tools to improve cancer identification and guide endoscopic resection over standard WLE assessment (8–10). Narrow-band imaging (NBI), using a narrowed bandwidth filter (specific blue and green wavelengths), can accentuate structural mucosal patterns and mucosal/submucosal vessels (8). Endocytoscopy (EC) with ultra-high magnification enables *in vivo* observation of both structural and cellular atypia during routine endoscopic examination (9). Confocal laser endomicroscopy (CLE) is another new enhanced technology. CLE can magnify mucosa up to 1000 times at a subcellular level of resolution up to 250 µm below the mucosal surface using fluorescein as the imaging agent (10). Despite the enhanced images provided by these newly developed technologies, several limitations remain in their applications. For NBI, in some cases, it is not easy to distinguish inflammatory lesions from tumor lesions, thereby contributing to high false-positive rates (11). For EC, the procedure time is prolonged and the recognition theory system has yet to be perfected (12). For CLE, fluorescein stains equally all cell types (including normal cells and cancer cells), and a real-time image version to differentiate cancer may be a great challenge (10).

CLE using a fluorescent-labeled molecular probe has been reported in order to identify colonic dysplasia and cancer (13). Herein, we also attempted to develop a new imaging approach to increase the diagnostic efficacy of the available imaging skills by employing a new fluorescein imaging agent differentially expressed in normal and tumor lesions. Previous studies have identified that colonic cancer has different fucosylation levels than the normal colon (14). Fucosylation is a type of glycosylation modification that adds fucose (6-deoxy-L-galactose) to oligosaccharides/proteins (15). It has been reported that in the absence of fucosylation, dysplasia appeared and proceeded to cancer mainly influencing the right colon and cecum (14). Ulex europaeus agglutinin-I (UEA-I), which specifically binds to α1-2 fucose glycan, is specifically used to detect fucosylation levels (16).

Therefore, we investigated the expression level of UEA in normal intestinal epithelium and colon cancer, with the purpose of estimating fluorescently labeled UEA-fluorescein isothiocyanate (FITC) as an intestinal imaging agent for detecting colonic cancer with optical imaging.

## Materials and Methods

### Study Design

This study aimed to evaluate UEA as an endoscopic molecular imaging marker for colon cancer. The differential expression of α (1,2) fucosylation in normal colorectal epithelium and colorectal tumor specimens was first demonstrated by UEA-FITC staining in a mouse azoxymethane/dextran sodium sulfate (AOM/DSS)-induced colitis-associated colorectal cancer model. Fluorescently labeled UEA was investigated as a molecular imaging marker to identify colorectal cancer in mouse and human colonic specimens. UEA-FITC was administered to the intestinal mucosa, and FITC was detected using CLE.

In mice, an AOM/DSS-induced colitis-associated colorectal cancer model was used, and the detailed methods are provided below. For human colon specimens, all imaged colon specimens were selected on the basis of the endoscopic diagnosis of adenoma or adenocarcinoma. First, WLE was used to detect the susceptive lesions. Then, endocytoscopy and CLE were used to observe the details. All imaged specimens were biopsied and assessed by a pathologist who was blinded to the endoscopic results. For UEA-FITC imaging with CLE, analysis of biopsied regions was reliant on both CLE judgment and histopathology. Histopathological diagnosis was considered the gold standard. This study was approved by the Ethics Committee of Tongji Medical College, Huazhong University of Science and Technology. The study was conducted in accordance with the principles of the Declaration of Helsinki.

### Animal Experiment

Juvenile male C57BL/6J mice (7 ± 1 weeks old, body weight 20 ± 1 g, Beijing HFK Bio-Technology Co., LTD, China) were housed in individually ventilated cages (four animals per cage) at the SPF facility of Huazhong University of Science and Technology under controlled environmental conditions (temperature 22 ± 2°C; relative humidity 60%–70%) with free access to standard laboratory chow and tap water, and maintained under a regular 12/12-h light/dark cycle. All animal care and experimental procedures were approved by the Animal Care Ministry of Health and were performed in accordance with national and EU guidelines for the handling and use of experimental animals. Animal studies were reported in compliance with the ARRIVE guidelines.

The AOM/DSS-induced colitis-associated colorectal cancer model was developed as previously described (17). Briefly, male mice were treated with AOM (10 mg/kg). Five days later, the mice were feed with 1%–2% DSS for 7 days, followed by regular water drinking for 14 days. This cycle was repeated for three times. During the whole trial, body weight, diarrhea, and macroscopic bleeding of the mice were examined. At the end of the animal experiment, after overnight fasting, mice were sacrificed under anesthesia, and colorectums were collected for WLE, CE, and CLE observation.

### Endocytoscopy

All colonoscopic inspections were conducted by well-trained endoscopists (more than 1,500 colonoscopies) using an integrated-type EC (Olympus, Tokyo, Japan). This instrument has a single lens on its tip with a hand lever; the one-touch switch or the hand lever enables consecutively increasing magnification power from the conventional endoscopic image to ultra-magnification power, without changing the scope. This device has a 520-fold magnification power with a focus depth of 50 μm, which can image the gland duct lumens and the shape of nuclei in the epithelial superficial layer. The images were obtained using 0.05% crystal violet and 1% methylene blue. The intestinal mucosa of mice was examined *in vitro* because of its narrow lumen, and the human intestinal mucosa was observed *in vivo*. Uniformly sized fusiform nuclei and roundish lumens regularly represent normal mucosa. Slit-like smooth lumens and uniform fusiform or roundish nuclei indicate adenoma. Vague gland formation and agglomeration of distorted nuclei suggested adenocarcinoma (**Figures 2A** and **3A**).

### UEA-FITC Imaging With CLE

After examination by WLE and EC, UEA-FITC staining was used for CLE. Colorectums were washed with saline; then, UEA-FITC (1/50 mg/ml) and IgG-FITC (1/50 mg/ml) were incubated for 2–3 min. Tissue-bound UEA-FITC was detected using a clinical CLE system. This probe-based system (pCLE) (Cellvizio Endomicroscopy System; Mauna Kea Technologies, Paris, France) is commercially available and consists of a flexible miniprobe that may be introduced through the working channel of a standard endoscope. This device was used with a 2.8-mm fiber optic probe. The probe provided a field of view of 240 μm and acquired videos. A typical CLE image of stained normal mucosa displays a hexagonal, honeycomb appearance of vessel architecture along with regular, round luminal openings, surrounded by a homogeneous layer of epithelial cells. Irregular epithelium and villiform- or tubular-shaped crypts all indicate adenoma. Adenocarcinoma causes disorders and destruction of the glands. Videos were taken, and data from entire video sequences (12 frames/s, 10- to 20-s videos) were used for quantitation analysis. For mice, 10 videos from normal mice (*n* = 10), 19 videos of 19 adenoma specimens from mice with adenoma (*n* = 5), and 20 videos of 20 tumor specimens from mice with tumors (*n* = 5) were analyzed. For humans, 10 videos from normal humans (*n* = 10), six videos of six adenoma specimens from patients with adenoma (*n* = 6), and four videos of four tumor specimens from patients with tumors (*n* = 4) were analyzed. To validate the quantitative analysis, we set to cancel the automatic control function of the laser power when using the CLE system. After examination by CLE, representative lesions were biopsied for histopathological correlation.

### Statistical Analysis

Statistical analysis was performed using SPSS software. All data are presented as the mean (95% confidence interval). One-way analysis of variance tests were used to determine the significance of UEA expression levels in different groups. For the diagnostic accuracy of UEA-FITC using CLE, sensitivity was calculated as TP/(TP + FN) and specificity was calculated as TN/(TN + FP), where TP equaled the number of true-positive cases, FN equaled the number of false-negative cases, TN equaled the number of true-negative cases, and FP equaled the number of false-positive cases.

## Results

### UEA-FITC Expression and Distribution

**Figure 1:**
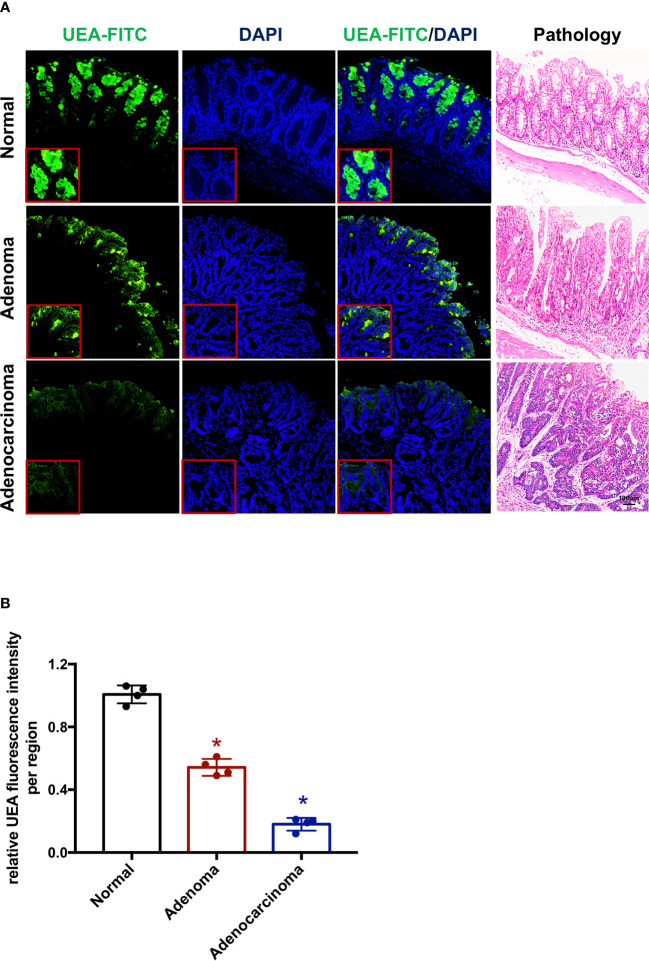
UEA-FITC expression and distribution in colorectal cancer. **(A)** Immunofluorescence of colorectum tissue sections showing UEA distribution in normal intestinal epithelium, adenoma, and cancer. The corresponding pathologic pictures were also shown. Scale bars, 250 μm. **(B)** Cancer/adenoma-to-normal fluorescence signal ratio of each colorectum (*n* = 4) imaged with immunofluorescence. Error bars, SD. *p < 0.05. UEA, ulex europaeus agglutinin; FITC, fluorescein isothiocyanate; SD, standard deviation.

Immunofluorescence was conducted to detect UEA-FITC in frozen mouse tissue sections of normal intestinal epithelium, adenoma, and colorectal tumor; however, the distribution was different (**Figure 1A**). The single layer of normal intestinal epithelium cells showed widely distributed UEA expression on the mucosal epithelial layer, whereas UEA expression on the mucosal epithelial layer decreased in cancer tissue. Furthermore, the UEA expression level of colorectal adenoma was between them both (**Figure 1B**). This difference in distribution indicates that UEA may be a feasible agent for the endoscopic molecular imaging of colorectal cancer.

### Endoscopic Molecular Imaging for Mice

**Figure 2:**
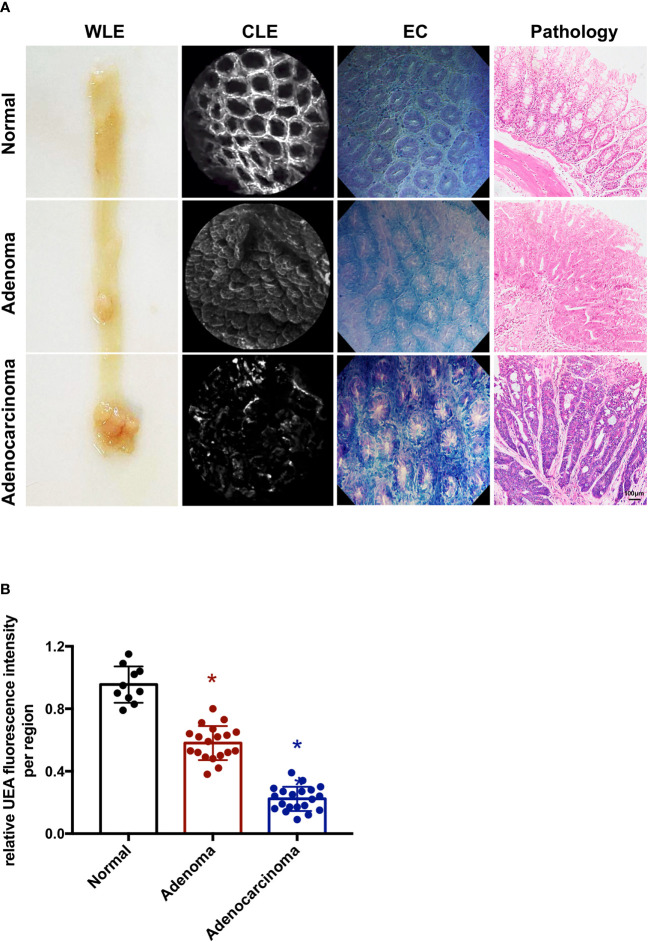
Endoscopic molecular imaging of mouse colorectal cancer using UEA-FITC and CLE. **(A)** Representative frames of WLE, CLE, and EC acquired from normal, adenoma, and cancer lesions, and the corresponding pathologic pictures. Scale bars, 100 μm. **(B)** Cancer/adenoma-to-normal fluorescence signal ratio of each colorectum (*n* = 4) imaged with CLE. Error bars, SD. *p < 0.05. UEA, ulex europaeus agglutinin; FITC, fluorescein isothiocyanate; WLE, white light endoscopy; CLE, confocal laser endomicroscopy; EC, endocytoscopy; SD, standard deviation.

An *ex vivo* endoscopic molecular imaging protocol was devised for mice (**Figure 2**). Fresh mouse colorectums were obtained from a mice AOM/DSS-induced colitis-associated colorectal cancer model (*n* = 10). For its narrow intestinal cavity, the colorectum was opened longitudinally for the sake of white light visual examination of the intestinal epithelium using a camera. After spraying 0.05% crystal violet plus 1% methylene blue onto the surface of a polyp or adenocarcinoma together with its surrounding normal mucosa, EC was used to observe the surface of the mucosa. Typical distinct EC images of stained normal mucosa, adenoma, and adenocarcinoma are shown in **Figure 2A**. Subsequently, a UEA labeled with fluorescein isothiocyanate (FITC) was instilled after washing the mucosa with saline. IgG-FITC was also used as a negative control (**Supplementary Figure 1**). Colorectums incubated with UEA-FITC and IgG-FITC were imaged using CLE. Normal, adenoma, or adenocarcinoma regions in each colorectum were imaged and biopsied. A pathologist was blinded to the imaging results and analyzed the histologic results. All colorectums were confirmed to have colorectal cancer on the basis of the histologic results. Representative frames of normal intestinal, adenoma, and adenocarcinoma lesions in each colorectum with corresponding histopathology taken by confocal videos are also shown in **Figure 2A**.

To quantitatively analyze the UEA-FITC signal in confocal videos, the average fluorescence intensity of all frames in the video was calculated as the mean fluorescence intensity. Videos from a normal region, adenoma region, and cancer (endoscopically diagnosed) were applied to determine the fluorescence intensity ratio of UEA-FITC coupling to cancer, adenoma, and normal tissue (**Figure 2B**). The results showed that the fluorescence intensity ratio of UEA-FITC in cancer tissues was significantly lower than that in normal tissues (*p* < 0.05).

### Endoscopic Molecular Imaging for Humans

**Figure 3:**
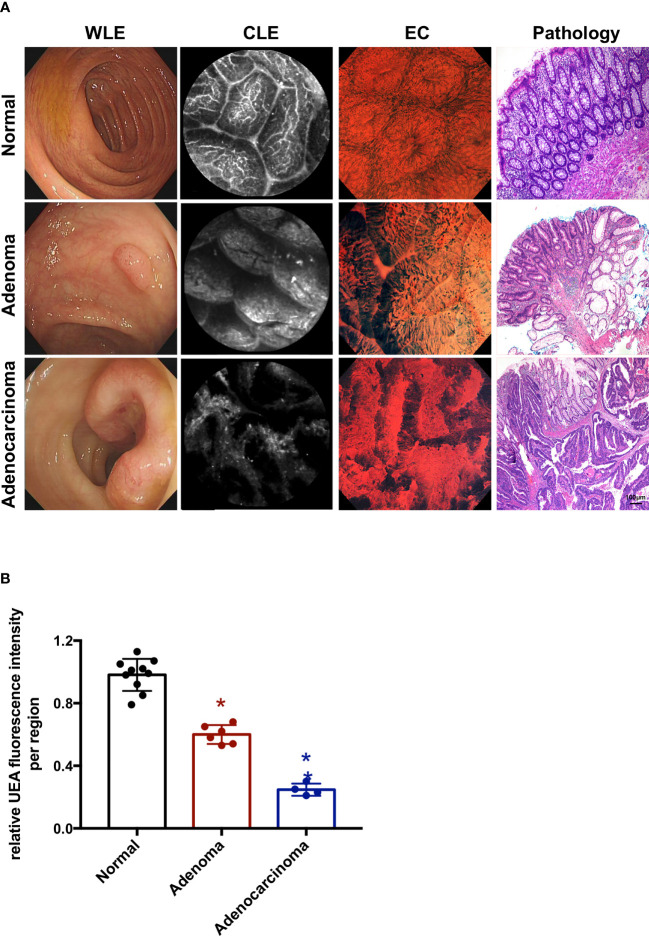
Endoscopic molecular imaging of human colorectal cancer using UEA-FITC and CLE. **(A)** Representative frames of WLE, CLE, and EC acquired from normal, adenoma, and cancer lesions, and the corresponding pathologic pictures. Scale bars, 100 μm. **(B)** Cancer/adenoma-to-normal fluorescence signal ratio of each colorectum (*n* = 4) imaged with CLE. Error bars, SD. *p < 0.05. UEA, ulex europaeus agglutinin; FITC, fluorescein isothiocyanate; WLE, white light endoscopy; CLE, confocal laser endomicroscopy; EC, endocytoscopy; SD, standard deviation.

For human colon specimens, all specimens were selected on the basis of endoscopic diagnosis of adenoma or adenocarcinoma (*n* = 10). The adenoma and adenocarcinoma lesions were removed endoscopically by EMR, ESD, or by surgery. The observation procedure for human colorectums was the same as that for mouse colorectums. The colorectums were first investigated by WLE and EC, and then by CLE. After incubation with UEA-FITC, fiber optic probe was done. IgG-FITC was used for the negative control (**Supplementary Figure 1**). The lesions were imaged and evaluated by a pathologist who was blinded to the imaging results. All 10 colorectal tumors were pathologically confirmed to have adenomas or cancers by histology. Representative frames from confocal videos taken of adenoma or adenocarcinoma lesions and para-cancer normal tissues with corresponding histopathology are shown in **Figure 3A**. The average fluorescence intensity of all frames in a confocal video was determined so as to quantitatively analyze the UEA-FITC signal. For each colorectum, videos from a normal region, adenoma region, and cancer were obtained to calculate the fluorescence intensity ratio of UEA-FITC coupling to cancer, adenoma, and normal tissue (**Figure 3B**). The results showed that compared to normal tissue, the fluorescence intensity ratio of UEA-FITC in cancer was significantly lower (*p* < 0.05).

### Diagnostic Accuracy of UEA-Targeted Imaging

Confocal images, including mouse and human specimens, were used prospectively to predict the histopathology. Overall, 69 different locations in 20 colorectums, including 20 locations from normal mucosa, 25 locations from adenoma, and 24 locations from cancer, were analyzed using a blind method. CLE was applied to evaluate all sites before biopsy, and histological results were compared with CLE judgment. The results of CLE judgment and histology are displayed in **Table 1**. The sensitivity and specificity of CLE for malignant lesion detection were 95.6% and 97.7%, respectively. The positive and negative predictive values were 91.6% and 95.6%, respectively. The overall correction rate for all sites was 95.6% by the CLE.

**Table 1:** Correlation between confocal imaging and histology.

Confocal diagnosis	Sites	Histology		
		Normal	Adenoma	Adenocarcinoma
Normal	20	20	0	0
Adenoma	25	2	22	1
Adenocarcinoma	24	0	2	22

## Discussion

The enhancement of endoscopic detection of colorectal cancer can increase the efficiency of tumor excision and decrease cancer recurrence and progression. Here, we show three main novel findings: (1) UEA is highly expressed in the normal intestinal epithelium but less in colorectal cancer, (2) the combination of UEA-FITC and CLE helped to identify patients with colorectal cancer, and (3) the real-time assessment by CLE conducted on UEA-FITC-targeted locations has a good diagnostic accuracy for colorectal cancer (sensitivity 96.4% and specificity 97.5%).

Previous studies have identified that colonic cancer has different fucosylation levels compared to normal colons (14). It has been reported that α1-3/4 fucosylation in the sera of colon cancer patients was higher than that in other groups (18). However, another study demonstrated that in the deficiency of fucosylation, dysplasia appeared and progressed to adenocarcinoma, mainly affecting the right colon and cecum (14). UEA-I, which combines with α1-2 fucose glycan, is usually used to detect α1-2 fucosylation levels. Our present study demonstrated decreased UEA levels in colonic cancer compared to normal colon tissue (**Figure 1**). This difference in distribution indicates that UEA may be a feasible agent for the endoscopic molecular imaging of colorectal cancer.

UEA-I is a lectin that specifically couples with α-linked fucose (16). Lectins are highly stereospecific proteins that can recognize numerous sugar structures and interact with glyco-conjugate complexes to form reversible bindings (19). Above all, they are abundantly discovered in plants and animals. Therefore, the UEA-I used here is a safe protein that is suitable for human use, although in our study, it was used *in vitro*. Furthermore, as the colorectum is a cavity organ, UEA-I can be administered locally without significant systemic absorption. Therefore, UEA-I is a viable and safe target for molecular imaging. The organ imaging protocol developed here facilitates the clinical use of UEA-targeted imaging for the detection and resection of colorectal cancer.

CLE combined with UEA-FITC can reveal differences in the morphology of the staining pattern. As shown in **Figures 2A** and **3A**, a typical CLE image of stained normal mucosa displays a hexagonal, honeycomb appearance of vessel architecture along with regular, round luminal openings, surrounded by a homogeneous layer of epithelial cells. Irregular epithelium and villiform- or tubular-shaped crypts all indicate adenoma. Adenocarcinoma causes disorders and destruction of the glands. These different morphologies could help to diagnose normal, adenoma, and adenocarcinoma lesions in the first step.

In addition, CLE combined with UEA-FITC was investigated for the digital nature of the confocal images, allowing for semi-quantitative analysis. *In vivo* endoscopic molecular imaging combining fluorescent agents and CLE has also been reported for esophageal lesions (20) and gastric dysplasia (21). In esophageal applications, the addition of a 3-biomarker panel (cyclin A, p53, and aneuploidy) led to a 50% reduction in the false-positive rate of CLE, obtaining a sensitivity of 89.2% and a specificity of 88.9% for any grade of dysplasia, respectively (20). In the gastric study, targeted H-ferritin was demonstrated in resected human early gastric cancers with good diagnostic efficacy (100% sensitivity and 90% specificity) using CLE (21). In previous studies, quantitation was determined using images obtained from video sequences (20, 21). In this study, data of normal and cancer regions collected from the whole confocal video sequences (12 frames/s, 10- to 20-s videos) were incorporated for analysis. We measured the UEA-FITC fluorescence intensity ratio of tumor tissue to normal tissue using this unbiased method (**Figures 2B** and **3B**).

Although CLE allowed for quantitation, the small field of view impeded efficient examination of the entire colon. WLE was first used to observe the entire colon, then EC was applied to detect suspicious lesions, and CLE was subsequently used. The combination of UEA-FITC and CLE has a good diagnostic accuracy for colorectal cancer (sensitivity 95.6% and specificity 97.7%), which is quite higher than the data reported on CLE alone (sensitivity 81%–91.4% and specificity 76%–85.7%) (22, 23). Estimating the benign and malignant lesions and determining the boundary of the malignant lesions are the key points for the diagnosis and treatment of colorectal cancer. The combination of UEA-FITC and CLE may help improve colorectal cancer detection and the thoroughness of tumor resection.

This study had several limitations. First, this study was limited to an *ex vivo* investigation, as required by the local ethics committee. However, despite the small number of human specimens, given the experience of our *ex vivo* human investigation, we are confident that topical administration *in vivo* of UEA-FITC would highlight adenoma and adenocarcinoma lesions in patients as well. Secondly, the relatively high cost and long learning curve of the procedure make it difficult for clinical use. Nevertheless, with the development of the economy and technology, we believe that it will be feasible in the near future. Thirdly, the actual advantages with respect to histology as the gold standard made this new approach more reliable, but it still could provide very reliable information.

In summary, we provide new concepts for identifying and validating molecular imaging targets for colorectal cancer. UEA can act as a cancer-specific imaging agent to improve the detection and treatment of CRC. The present imaging approach utilizes the simplicity of accessing the colorectal lumen, the well-established intraintestinal route for administering drugs in the colorectum, and the availability of clinical level imaging tools involving EC and CLE. We demonstrate that this method is feasible and may be adaptable for further development and validation of imaging agents in other hollow organs. Together, this research provides evidence for UEA as a colorectal cancer imaging target with the potential for clinical translation.

## Data Availability Statement

The original contributions presented in the study are included in the article/**Supplementary Material**. Further inquiries can be directed to the corresponding author.

## Ethics Statement

The studies involving human participants were reviewed and approved by the Ethics Committee of Tongji Medical College, Huazhong University of Science and Technology, Affiliated to the Huazhong University of Science and Technology. Written informed consent for participation was not required for this study in accordance with the national legislation and the institutional requirements. The animal study was reviewed and approved by Ethics Committee of Tongji Medical College, Huazhong University of Science and Technology, Affiliated to the Huazhong University of Science and Technology. Written informed consent was not obtained from the individual(s) for the publication of any potentially identifiable images or data included in this article.

## Author Contributions

WW and ST: Study design, manuscript preparation, statistical analysis, and study coordinator. XJ and SP: Animal experiment and data collection. HS, MF, and WJ: Patient experiment and data collection. ZW and WH: Animal data acquisition, and data analysis and interpretation. XX: Revising the manuscript. RL: Study design, manuscript revision, study supervision, and foundation support. All authors contributed to the article and approved the submitted version.

## Funding

This work was supported by the National Natural Science Foundation of China (Nos. 81900580, 81974068, and 81770539), the Natural Science Foundation of Hubei Province (No. 2017CFA061), the Non-profit Central Research Institute Fund of Chinese Academy of Medical Sciences (2020-PT330-003), and the Open Research Fund of NHC Key Laboratory of Prevention and Treatment of Central Asia High Incidence Diseases.

## Conflict of Interest

The authors declare that the research was conducted in the absence of any commercial or financial relationships that could be construed as a potential conflict of interest.

## Publisher’s Note

All claims expressed in this article are solely those of the authors and do not necessarily represent those of their affiliated organizations, or those of the publisher, the editors and the reviewers. Any product that may be evaluated in this article, or claim that may be made by its manufacturer, is not guaranteed or endorsed by the publisher.

## Supplementary Material

Supplementary Figure 1 Endoscopic molecular imaging of colorectal cancer using IgG-FITC and CLE in mouse and human.

Supplementary Video 1 A video of CLE combined with UEA to detect normal colon tissue in mice CLE, confocal laser endomicroscopy; UEA, Ulex europaeus agglutinin.

Supplementary Video 2 A video of CLE combined with UEA to detect colon adenoma tissue in mice CLE, confocal laser endomicroscopy; UEA, Ulex europaeus agglutinin.

Supplementary Video 3 A video of CLE combined with UEA to detect colorectal cancer in mice CLE, confocal laser endomicroscopy; UEA, Ulex europaeus agglutinin.

## References

[1] Chiu PWY Uedo N Singh R Gotoda T Ng EKW Yao K. An Asian Consensus on Standards of Diagnostic Upper Endoscopy for Neoplasia. Gut (2019) 68:186–97. doi: 10.1136/gutjnl-2018-317111 30420400

[2] van der Sommen F Curvers WL Nagengast WB. Novel Developments in Endoscopic Mucosal Imaging. Gastroenterology (2018) 154:1876–86. doi: 10.1053/j.gastro.2018.01.070 29462601

[3] Arnold M Sierra MS Laversanne M Soerjomataram I Jemal A Bray F. Global Patterns and Trends in Colorectal Cancer Incidence and Mortality. Gut (2017) 66:683–91. doi: 10.1136/gutjnl-2015-310912 26818619

[4] Bray F Ferlay J Soerjomataram I Siegel RL Torre LA Jemal A. Global Cancer Statistics 2018: GLOBOCAN Estimates of Incidence and Mortality Worldwide for 36 Cancers in 185 Countries. CA Cancer J Clin (2018) 68:394–424. doi: 10.3322/caac.21492 30207593

[5] Siegel RL Miller KD Goding Sauer A Fedewa SA Butterly LF Anderson JC. Colorectal Cancer Statistics, 2020. CA Cancer J Clin (2020) 70(3):145–64. doi: 10.3322/caac.21601 32133645

[6] Ferlitsch M Moss A Hassan C Bhandari P Dumonceau JM Paspatis G. Colorectal Polypectomy and Endoscopic Mucosal Resection (EMR): European Society of Gastrointestinal Endoscopy (ESGE) Clinical Guideline. Endoscopy (2017) 49:270–97. doi: 10.1055/s-0043-102569 28212588

[7] Benson AB 3rd Venook AP Cederquist L Chan E Chen YJ Cooper HS. Colon Cancer, Version 1.2017, NCCN Clinical Practice Guidelines in Oncology. J Natl Compr Canc Netw (2017) 15:370–98. doi: 10.6004/jnccn.2017.0036 28275037

[8] Atkinson NSS Ket S Bassett P Aponte D De Aguiar S Gupta N. Narrow-Band Imaging for Detection of Neoplasia at Colonoscopy: A Meta-Analysis of Data From Individual Patients in Randomized Controlled Trials. Gastroenterology (2019) 157:462–71. doi: 10.1053/j.gastro.2019.04.014 30998991

[9] Kumagai Y Takubo K Kawada K Higashi M Ishiguro T Sobajima J. A Newly Developed Continuous Zoom-Focus Endocytoscope. Endoscopy (2017) 49:176–80. doi: 10.1055/s-0042-11926 27842421

[10] Wanders LK East JE Uitentuis SE Leeflang MM Dekker E. Diagnostic Performance of Narrowed Spectrum Endoscopy, Autofluorescence Imaging, and Confocal Laser Endomicroscopy for Optical Diagnosis of Colonic Polyps: A Meta-Analysis. Lancet Oncol (2013) 14:1337–47. doi: 10.1016/S1470-2045(13)70509-6 24239209

[11] van den Broek FJ Fockens P van Eeden S Reitsma JB Hardwick JC Stokkers PC. Endoscopic Tri-Modal Imaging for Surveillance in Ulcerative Colitis: Randomised Comparison of High-Resolution Endoscopy and Autofluorescence Imaging for Neoplasia Detection; and Evaluation of Narrow-Band Imaging for Classification of Lesions. Gut (2008) 57:1083–9. doi: 10.1136/gut.2007.144097 PMC2564833 18367559

[12] Ichimasa K Kudo SE Mori Y Wakamura K Ikehara N Kutsukawa M. Double Staining With Crystal Violet and Methylene Blue is Appropriate for Colonic Endocytoscopy: An *In Vivo* Prospective Pilot Study. Dig Endosc (2014) 26:403–8. doi: 10.1111/den.12164 PMC4232925 24016362

[13] De Palma GD Colavita I Zambrano G Giglio MC Maione F Luglio G. Detection of Colonic Dysplasia in Patients With Ulcerative Colitis Using a Targeted Fluorescent Peptide and Confocal Laser Endomicroscopy: A Pilot Study. PloS One (2017) 12:e0180509. doi: 10.1371/journal.pone.0180509 28666016 PMC5493408

[14] Wang Y Huang D Chen KY Cui M Wang W Huang X. Fucosylation Deficiency in Mice Leads to Colitis and Adenocarcinoma. Gastroenterology (2017) 152(1):193–205 e10. doi: 10.1053/j.gastro.2016.09.004 27639802 PMC5164974

[15] Goto Y Uematsu S Kiyono H. Epithelial Glycosylation in Gut Homeostasis and Inflammation. Nat Immunol (2016) 17(11):1244–51. doi: 10.1038/ni.3587 27760104

[16] Tian R Zhao S Liu G Chen H Ma L You H. Construction of Lanthanide-Doped Upconversion Nanoparticle-Uelx Europaeus Agglutinin-I Bioconjugates With Brightness Red Emission for Ultrasensitive *In Vivo* Imaging of Colorectal Tumor. Biomaterials (2019) 212:64–72. doi: 10.1016/j.biomaterials.2019.05.010 31103947

[17] Isermann T Şener ÖÇ Stender A Klemke L Winkler N Neesse A. Suppression of HSF1 Activity by Wildtype P53 Creates a Driving Force for P53 Loss-of-Heterozygosity. Nat Commun (2021) 12(1):4019. doi: 10.1038/s41467-021-24064-1 34188043 PMC8242083

[18] Park SY Lee SH Kawasaki N Itoh S Kang K Hee Ryu S. Alpha1-3/4 Fucosylation at Asn 241 of Beta-Haptoglobin is a Novel Marker for Colon Cancer: A Combinatorial Approach for Development of Glycan Biomarkers. Int J Cancer (2012) 130(10):2366–76. doi: 10.1002/ijc.26288 21780104

[19] Mishra A Behura A Mawatwal S Kumar A Naik L Mohanty SS. Structure-Function and Application of Plant Lectins in Disease Biology and Immunity. Food Chem Toxicol (2019) 134:110827. doi: 10.1016/j.fct.2019.110827 31542433 PMC7115788

[20] di Pietro M Bird-Lieberman EL Liu X Nuckcheddy-Grant T Bertani H O'Donovan M. Autofluorescence-Directed Confocal Endomicroscopy in Combination With a Three-Biomarker Panel Can Inform Management Decisions in Barrett's Esophagus. Am J Gastroenterol (2015) 110(11):1549–58. doi: 10.1038/ajg.2015.295 26416188

[21] Du Y Fan K Zhang H Li L Wang P He J. Endoscopic Molecular Imaging of Early Gastric Cancer Using Fluorescently Labeled Human H-Ferritin Nanoparticle. Nanomedicine (2018) 14:2259–70. doi: 10.1016/j.nano.2018.07.007 30056091

[22] Shahid MW Buchner AM Raimondo M Woodward TA Krishna M Wallace MB. Accuracy of Real-Time vs. Blinded Offline Diagnosis of Neoplastic Colorectal Polyps Using Probe-Based Confocal Laser Endomicroscopy: A Pilot Study. Endoscopy (2012) 44(7):343–8. doi: 10.1055/s-0031-1291589 22382851

[23] Andre B Vercauteren T Buchner AM Krishna M Ayache N Wallace MB. Software for Automated Classification of Probe-Based Confocal Laser Endomicroscopy Videos of Colorectal Polyps. World J Gastroenterol (2012) 18(39):5560–9. doi: 10.3748/wjg.v18.i39.5560 PMC3482642 23112548

