# Supplementary figures and images for: Molecular Imaging of Ulex Europaeus Agglutinin in Colorectal Cancer Using Confocal Laser Endomicroscopy (With Video)
Source: Front Oncol. 2021 Dec 20;11:792420. doi: 10.3389/fonc.2021.792420 (PMC8722710; doi:10.3389/fonc.2021.792420)

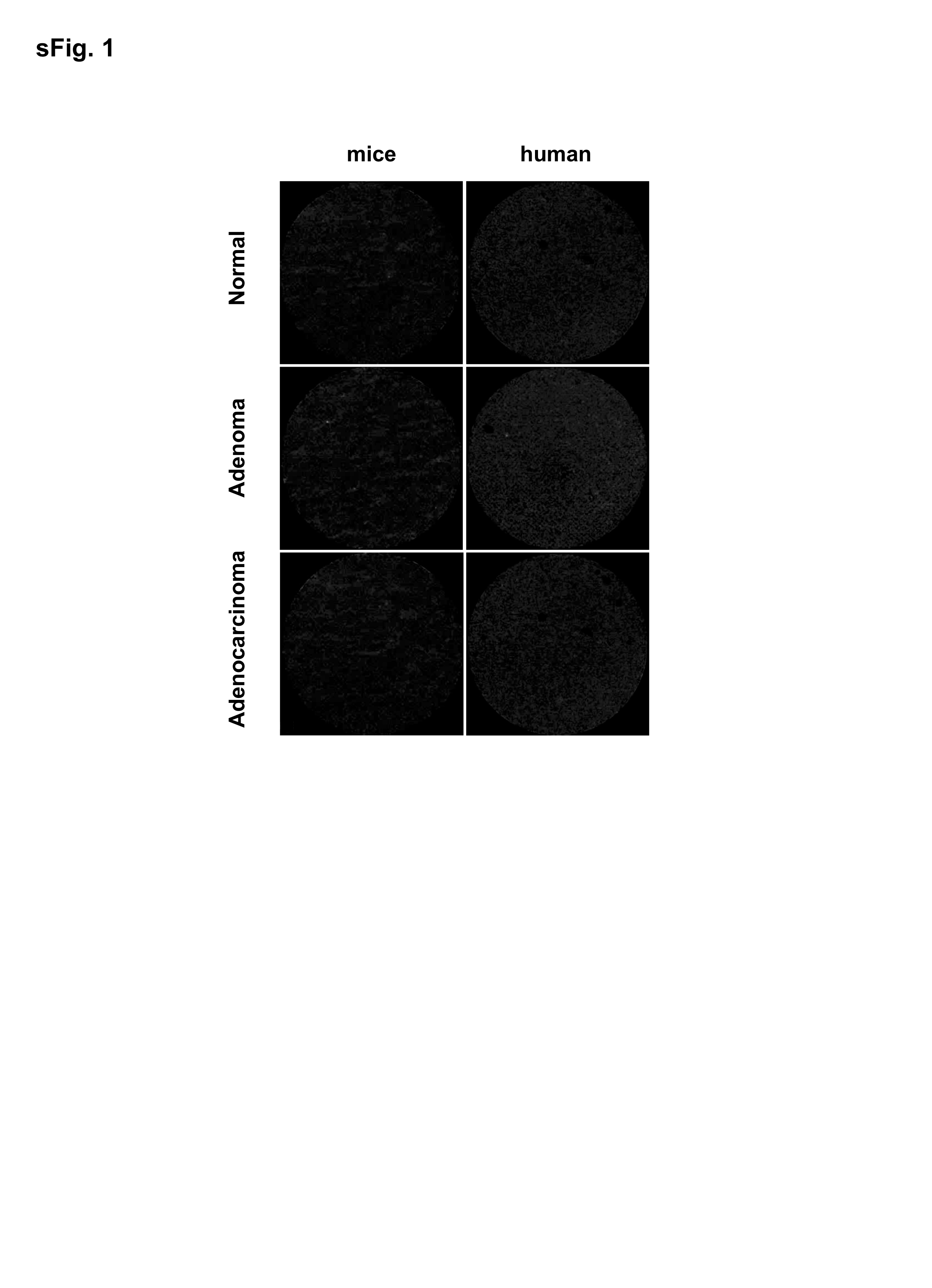

Supplement: Supplementary Figure 1 — Endoscopic molecular imaging of colorectal cancer using IgG-FITC and CLE in mouse and human. [file Image_1.jpeg]
